# Electrical Stimulation Degenerated Cochlear Synapses Through Oxidative Stress in Neonatal Cochlear Explants

**DOI:** 10.3389/fnins.2019.01073

**Published:** 2019-10-14

**Authors:** Qiong Liang, Na Shen, Bin Lai, Changjian Xu, Zengjun Sun, Zhengmin Wang, Shufeng Li

**Affiliations:** ^1^Department of Otolaryngology, Eye and ENT Hospital of Fudan University, Shanghai, China; ^2^National Health Commission Key Laboratory of Hearing Medicine, Shanghai, China; ^3^Department of Otolaryngology, Zhongshan Hospital of Fudan University, Shanghai, China; ^4^State Key Laboratory of Medical Neurobiology, Shanghai, China; ^5^Shanghai Cochlear Engineering Technology Research Center, Shanghai, China

**Keywords:** cochlear explants, electrical stimulation, oxidative stress, calcium influx, ebselen, synapses, spiral ganglion neuron

## Abstract

Neurostimulation devices use electrical stimulation (ES) to substitute, supplement or modulate neural function. However, the impact of ES on their modulating structures is largely unknown. For example, recipients of cochlear implants using electroacoustic stimulation experienced delayed loss of residual hearing over time after ES, even though ES had no impact on the morphology of hair cells. In this study, using a novel model of cochlear explant culture with charge-balanced biphasic ES, we found that ES did not change the quantity and morphology of hair cells but decreased the number of inner hair cell (IHC) synapses and the density of spiral ganglion neuron (SGN) peripheral fibers. Inhibiting calcium influx with voltage-dependent calcium channel (VDCC) blockers attenuated the loss of SGN peripheral fibers and IHC synapses induced by ES. ES increased ROS/RNS in cochlear explants, but the inhibition of calcium influx abolished this effect. Glutathione peroxidase 1 (GPx1) and GPx2 in cochlear explants decreased under ES and ebselen abolished this effect and attenuated the loss of SGN peripheral fibers. This finding demonstrated that ES induced the degeneration of SGN peripheral fibers and IHC synapses in a current intensity- and duration-dependent manner *in vitro*. Calcium influx resulting in oxidative stress played an important role in this process. Additionally, ebselen might be a potential protector of ES-induced cochlear synaptic degeneration.

## Introduction

Neurostimulation devices, for example visual prosthetics, auditory prosthetics, deep brain stimulation device, prosthetics for pain relief, motor prosthetics and brain-computer interfaces, are promising therapeutics for neurological disorders by supplanting or supplementing the input and/or output of the nervous system. These devices were initially designed to bypass neural deficits that occurred as a result of injuries or diseases. Currently, neurostimulation devices are even developed to modulate existing neural function to improve performance, especially in the application of future brain-computer interfaces. Cochlear implants (CIs) are the most widely used neural prosthetic. Traditional CIs restore hearing perception by delivering electrical signals converted from sound information to spiral ganglion neurons (SGNs), bypassing the defective or missing mechanosensory structures of the organ of Corti, i.e., hair cells. In the last decade, electric-acoustic stimulation (EAS) technology was newly developed for patients with severe or profound high-frequency hearing loss and residual low-frequency hearing (Von Ilberg et al., 1999; Gantz and Turner, 2003; Kiefer et al., 2005). This technology uses a short electrode array in the basal to middle part of the cochlear duct, leaving the apical part intact to preserve the residual low-frequency hearing. Patients are then able to receive acoustic signals at the apical part of the cochlea and electrical stimulation (ES) at the basal and middle part of the cochlea, simultaneously. Compared to full-insertion CI, EAS technology significantly improves music appreciation and speech recognition in background noise (Turner et al., 2004, 2008; Gfeller et al., 2006). Accordingly, the preservation of residual low-frequency hearing is critical to EAS recipients. Unfortunately, clinical trials showed that 30–75% of EAS recipients experienced delayed progressive loss of residual low-frequency hearing over time after the activation of EAS (Gantz et al., 2009; Gstoettner et al., 2009; Santa Maria et al., 2013). Understanding how existing hearing function deteriorates under EAS might benefit not only the preservation of the residual hearing of EAS recipients but also the protection of existing neural functions on which, other neurostimulation devices depend. However, the mechanism of this delayed hearing impairment is largely unknown. Animal studies suggested that reduced endocochlear potential due to lateral wall or stria vascularis damage (Wright and Roland, 2013) and disturbed traveling wave due to fibrosis or new bone growth (Choi and Oghalai, 2005) were associated with the hearing loss of EAS recipients. Nevertheless, there is still a lack of strong evidence to support these theories. Previous animal studies demonstrated that ES did not cause any morphological changes in hair cells or SGNs (Ni et al., 1992; Shepherd et al., 1994; Coco et al., 2007; O’Leary et al., 2013). Notably, to the best of our knowledge, the status of synapses between SGNs and inner hair cells (IHCs) in EAS-induced hearing loss has not yet been investigated. However, the loss of IHC synapses has been shown to play an important role in noise-induced hearing loss (Kujawa and Liberman, 2009; Lin et al., 2011) and in age-related hearing loss (Makary et al., 2011; Sergeyenko et al., 2013).

Cochlear implants use charge-balanced biphasic pulses to stimulate SGNs. The depolarization of the SGN membrane caused by ES results in calcium influx through various types of voltage-dependent calcium channels (VDCCs). Excessive calcium influx could lead to the injuries of SGN (Hegarty et al., 1997; Roehm et al., 2008) and hair cells (Fridberger et al., 1998). Oxidative stress also plays important roles in hearing loss induced by noise, aminoglycoside antibiotics, cisplatin and aging (Choi and Choi, 2015; Sheth et al., 2017; Tavanai and Mohammadkhani, 2017). We postulated that excessive calcium influx through VDCCs and the resulting increase in oxidative stress might be involved in the loss of residual hearing due to chronic ES.

In this study, we used cochlear explants culture with ES of charge-balanced biphasic pulses to investigate the impact of ES on SGN peripheral fibers, hair cells and their synapses. We demonstrated that CI with ES could induce the degeneration of IHC synapses and SGN peripheral fibers through calcium influx and resulting oxidative stress.

## Materials and Methods

### Cochlear Explant Culture

All procedures were approved by the Ethics Review Board of Eye and ENT Hospital of Fudan University (No. 2013024). Sprague Dawley rat pups of 4–6 postnatal days old of both sexes were provided by Shanghai SIPPR-Bk Lab Animal Co., Ltd. The cochlear explant culture was previously used to investigate the excitotoxic damage of IHC-SGN synapses (Wang and Green, 2011). Briefly, the cochlea were dissected out in ice-cold PBS. The osseous labyrinth, stria vascularis and spiral ligament were carefully removed. With the organ of Corti and modiolus preserved intact, Reissner’s membrane and tectorial membrane were carefully removed with fine forceps. After the upper and basal turns were cut off, the middle turns were cut into small pieces and plated on poly-L-lysine-treated chamber slides. We usually dissected 5 pups and collected 10 cochleae at one time. Then the middle parts of cochlear tissues were pooled together and each of them was cut into 3–4 small pieces. Six pieces of cochlear tissues were then randomly put into each chamber. Unless otherwise indicated, the explants during the whole experiments, were maintained in a 37°C humidified incubator with 5% CO_2_ and in high glucose Dulbecco’s modified eagle’s medium (DMEM, Life Technologies, 11965) with N2 supplement (Life Technologies, 17502-048), 10% fetal bovine serum (Gibco, 10099-141), 10 μg/ml insulin (Sigma-Aldrich, I6634), 50 ng/ml neurotrophin-3 (NT-3, Sigma-Aldrich, N1905) and 50 ng/ml brain-derived neurotrophic factor (BDNF, Sigma-Aldrich, B3795). The explants were first allowed to settle down on the chamber floor for 24 h before the following treatments. The floating explants were discarded and the adherent ones were used for the following experiments.

### Chamber Slide With ES

**FIGURE 1:**
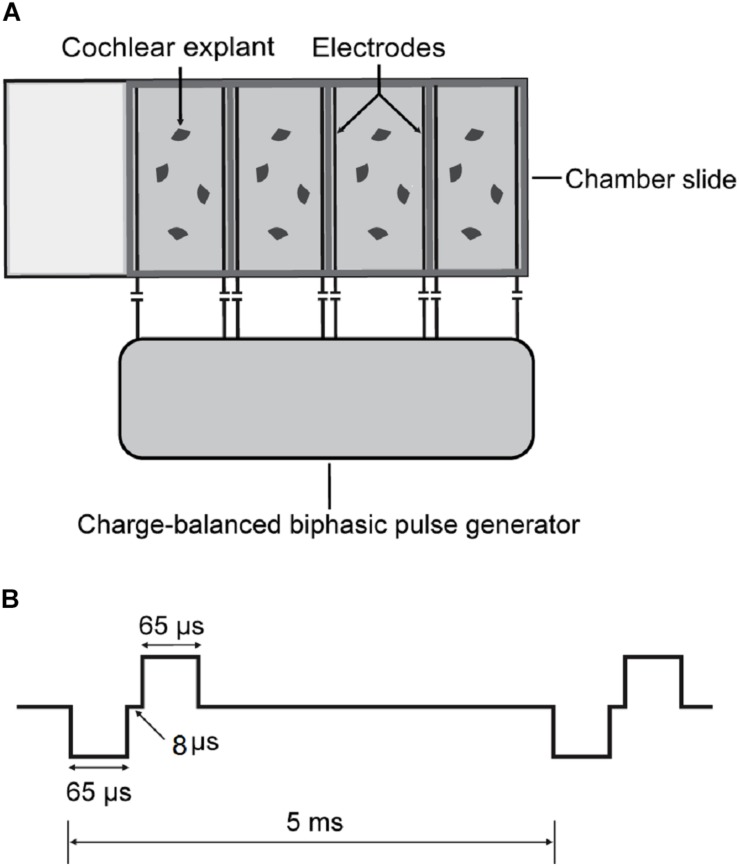
Schematics of cochlear explant culture under charge-balanced biphasic ES. **(A)** Four-well chamber slides were used in cochlear explant culture. Two parallel platinum-iridium wires were introduced into each chamber against the floor through four holes at four corners. The wires were then connected with a charge-balanced biphasic pulse generator. **(B)** The charge-balanced biphasic pluses for ES had adjustable amplitudes, a 65-μs pulse width, an 8-μs open-circuit interphase gap, a 4862-μs short-circuit phase, and a 200 Hz frequency.

To investigate the impact of ES on cochlear structures, we established a culture system of cochlear explants under ES (Figure 1). Briefly, two parallel platinum-iridium wires were introduced into a four-well chamber slide system (154526, Thermo Scientific) through four holes at four corners against the chamber floor. The holes were sealed with silicon glue to secure the wires which were connected to a multichannel charge-balanced biphasic pulse generator (Listent Medical Tech Co., Ltd.). The charge-balanced biphasic pulses used for ES held adjustable amplitudes with a 65-μs pulse width, 8-μs open-circuit interphase gap, and 4862-μs short-circuit phase at a frequency of 200 Hz. The distance between the two paralleling wires was 1 cm and the volume of culture medium in each chamber was 0.6 ml. The maximum charge density used in this study was 0.043 μC/cm^2^/phase when a maximum current intensity of 400 μA was used. This charge intensity was far less than 15 to 65 μC/cm^2^/phase which was suggested as the maximum level of charge intensity in commercial CIs (Zeng et al., 2008).

### Application of VDCC Blocker, Ebselen and H_2_O_2_

Various VDCC blockers, ebselen (40 μM; Sigma, E3520) and H_2_O_2_ (0.25 mM; Aladdin, H112517) were added to the culture medium. The VDCC blockers included the L-type channel blocker verapamil (VPL, 10 μM; Sigma, V4629), the N-type channel blocker ω–conotoxin GVIA (GVIA, 1 μM; Sigma, C9915), the P/Q-type channel blocker ω-agatoxin IVA (IVA, 1 μM; Sigma, A6719), the mixture of the three above blockers (CCBM, 10 μM VPL/1 μM GVIA/1 μM IVA), and the non-selective calcium channel blocker cadmium chloride (Cd, 10 μM; Sigma-Aldrich, 439800). For the calcium-free environment, the culture medium was completely replaced by calcium-free DMEM (Gibco, 21068028) with 1 mM EDTA, N2, BDNF, NT3, and insulin.

### Immunocytochemistry

Cochlear explant cultures were fixed with 4% paraformaldehyde for 15 min and permeabilized with 0.2% Triton X-100 and 10% donkey serum in PBS for 1 h. For immunostaining, the tissues were sequentially incubated with primary at 4°C overnight and with secondary antibodies for 1 h at room temperature diluted in PBS with 10% donkey serum. Primary antibodies were used as follows: anti-NF200 (1:400; Sigma, N0142) to label the SGNs and their peripheral fibers, anti-PSD95 (1:1000; Millipore, MABN68) to label postsynaptic densities (PSDs) in SGNs, and anti-Myo7A (1:800; Proteus BioSciences, 25-6790) or Alexa Fluor 647 phalloidin (1:200; Thermo Fisher Scientific, A22287) to label hair cells. Secondary antibodies were conjugated with Alexa Fluor 488, Alexa Fluor 546 and Alexa Fluor 647 (1: 800; Thermo Fisher Scientific).

### Measuring Reactive Oxygen Species (ROS)/Reactive Nitrogen Species (RNS) Activity

The total ROS/RNS activity was measured by a ROS/RNS Assay Kit (Cell Biolabs, STA-347-5) according to the provided procedure. Briefly, cochlear explant cultures under different conditions were removed and rapidly homogenized under ice-cold conditions. The homogenates were then centrifuged, and the supernatants were reacted with dichlorofluorescein in a DiOxyQ probe for spectrofluorimetric measurement.

### Real-Time PCR

For real-time PCR, PCR was conducted using an Applied Biosystems 7500 Real-time PCR System. Cochlear explants were harvested from cover slips and total RNA was purified with an RNeasy Plus Micro Extraction Kit (Qiagen, 74034). Then the RNA was reverse transcribed with a High Capacity RNA-to-cDNA kit (TaKaRa, RR036A) (Applied Biosystems, Foster City, CA, United States). The following primer pairs were designed using Primer3 software: β-actin, (F) CCTCTATGCCAACACAGT and (R) AGCCACCAATCCACACAG, with amplicon lengths of 155 bp; and glutathione peroxidase 2 (Gpx2), (F) AGACACTGGGAA ACCGAAGC and (R) AAGGAA ATGGGTGGCAGGAA, with amplicon lengths of 65 bp.

### Quantitative Analysis of SGN Peripheral Fibers, IHC Synapses and Hair Cells

**FIGURE 2:**
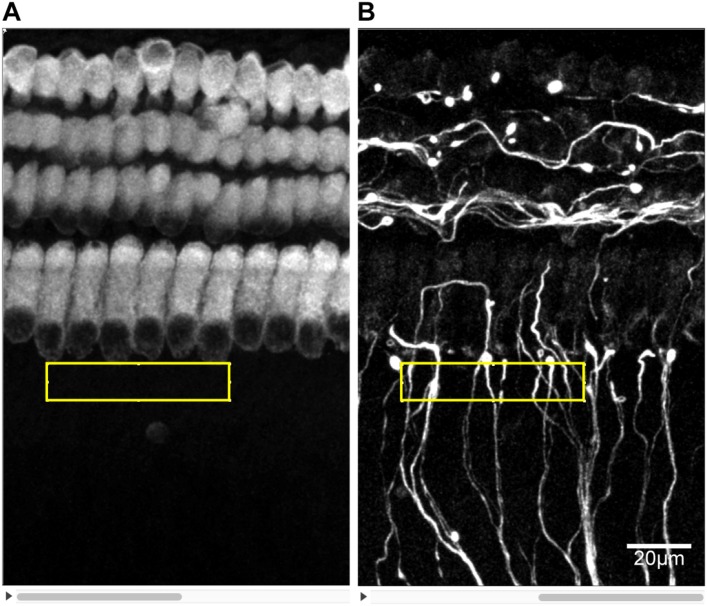
Measuring the density of SGN peripheral fibers. The z-stacks of PSD95 and NF200 8-bit grayscale images of the same area were prepared in ImageJ. **(A)** A certain rectangular area of 40 × 200 pixels was selected just against the base of IHCs in PSD95 images. **(B)** The mean grayscale of the same selected area in NF200 images minus that of a background area of the same size was determined as the density of SGN peripheral fibers.

Digital images of immunostained cochlear explants were acquired by a Leica SP8 confocal microscope. Serial images of each explant at a 0.3 μm interval (*z*-axis) were recorded to generate a z-stack of images that could be projected onto a single plane (z-projection). Images of hair cells, IHC synapses and SGN peripheral fibers were simultaneously obtained with a 60×, 1.5 numerical aperture objective, while hair cells and SGN peripheral fibers were scanned at 40×, in different experiments. Then, the images were analyzed with ImageJ software. The number of SGN-IHC synapses was determined by counting the numbers of PSD-95 puncta on IHCs and in contact with NF200-positive neurites slice by slice. Each puncta was counted in the first slice in which it appeared in focus to avoid being counted again. In the NF200 images, SGN peripheral nerve fibers in the area near the inner hair cell were crossing and overlapping. As a result, the fibers were hard to distinguish and count. We used the gray value of immunofluorescence in NF200 images to quantify the relative density of SGN peripheral nerve fibers. Images of each SGN peripheral fibers were captured using the same exposure time and light intensity and at the same sitting. At first, MYO7A and NF200 images from same location were converted to 8-bit grayscale images and constituted to a stack in ImageJ. A rectangle area with the dimension of 40 × 200 pixels was selected closely against to the base of inner hair cells in MYO7A images. That area coincided with the region that PSD-95 puncta distributed. Images of each SGN peripheral fibers were captured using the same exposure time and light intensity and at the same sitting. Then the mean gray value of the same area subtracted by that of background area in NF200 images was measured and determined as the relative density of SGN peripheral nerve fibers (Figure 2).

### Statistical Analysis

Statistical analysis was performed by GraphPad Prism 7 (GraphPad Software, Inc., CA, United States). Unless otherwise indicated, significances of differences among various conditions were compared by one-way ANOVA followed by Dunnett’s multiple comparisons test.

## Results

### ES Decreased the Quantity of SGN Peripheral Fibers and IHC Synapses but Not the Quantity of Hair Cells

**FIGURE 3:**
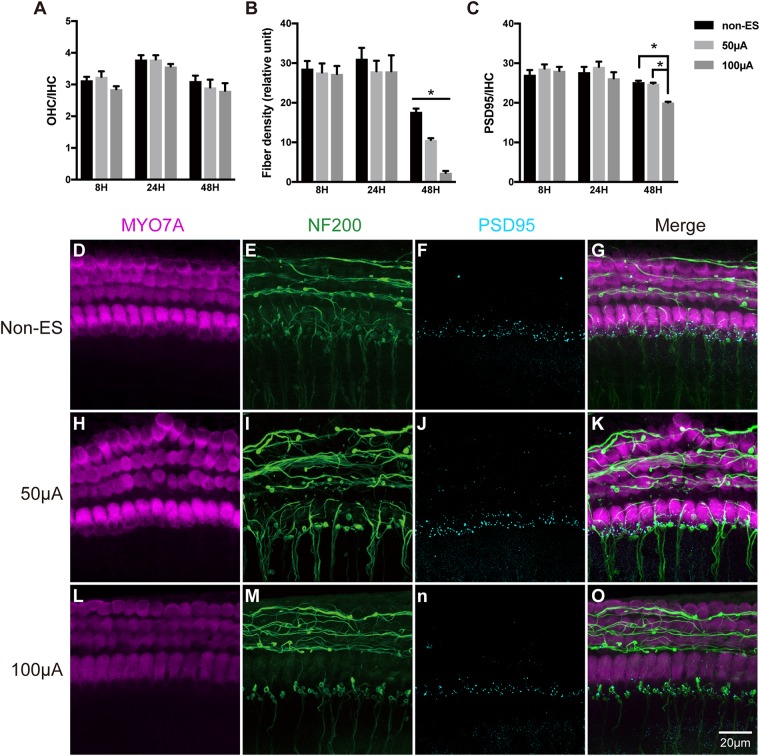
ES did not change the quantity and morphology of hair cells in cochlear explants but induced the loss of IHC synapses and SGN peripheral fibers. **(A)** After 8, 24 or 48 h of ES, the OHC/IHC ratio was comparable in cochlear explants without ES (non-ES group), under 50 μA ES and 100 μA ES (8 h/50 μA, *P* = 0.8955; 8 h/100 μA, *P* = 0.4851; 24 h/50 μA, *P* > 0.9999; 24h/100 μA, *P* = 0.5872; 48 h/50 μA, *P* = 0.6174 and 48 h/100 μA, *P* = 0.3631), *n* = 9–20 in each group. **(B)** The density of SGN peripheral fibers significantly decreased after 48 h/50 μA and 48 h/100 μA ES compared to the non-ES group (*P* = 0.0097, *P* < 0.0001, respectively), while the fiber density in explants after 8 h or 24 h ES was comparable to that in non-ES explants (8 h/50 μA, *P* = 0.9096; 8 h/100 μA, *P* = 0.8528; 24 h/50 μA, *P* = 0.4702; 24 h/100 μA, *P* = 0.4854), *n* = 9–20 in each group. **(C)** The PSD95/IHC ratio in explants with 48 h/100 μA ES was significantly different from that in non-ES explants (*P* < 0.0001), while PSD95/IHC ratio in explants with other treatments was comparable to that in non-ES explants (8 h/50 μA, *P* = 0.4526; 8 h/100 μA, *P* = 0.7005; 24 h/50 μA, *P* = 0.5011; 24 h/100 μA, *P* = 0.3921; 48 h/50 μA, *P* = 0.9170), *n* = 9–20 in each group. **(D–O)** Typical images of cochlear explants treated with 48 h/non-ES **(D–G)**, 48 h/50 μA ES **(H–K)**, and 48 h/100 μA ES **(L–O)**. The quantity and morphology of IHCs and OHCs (in magenta, labeled with anti-Myo7A) were comparable in explants treated with non-ES **(D)**, 50 μA ES **(H)** and 100 μA ES **(L)**. The density of SGN peripheral fibers (in green, labeled with anti-neurofilament-200, NF200) in explants treated with 50 μA (I) or 100 μA ES **(M)** was less than that in explants treated with non-ES **(E)**. The number of IHC synapses (in cyan, labeled with anti-PSD95) in explants treated with 100 μA ES **(N)** was much less than that in explants treated with 50 μA ES **(J)** or non-ES **(F)**. ^∗^*P* < 0.05. Data represent the mean + SEM. Two-way ANOVA followed by Dunnett’s multiple comparisons test was used in all the experiments mentioned above.

To investigate the impact of ES on cochlear structure, we cultured cochlear explants in a chamber slide system with multichannel charge-balanced biphasic pulse generators (Figure 1), which has been demonstrated in our previous work (Shen et al., 2016). The cochlear explants were electrically stimulated by charge-balanced biphasic electrical pulses with an amplitude of 50 or 100 μA amplitude for 8, 24, or 48 h. Cochlear explants cultured for the same duration and without ES were used as the control groups, respectively (non-ES group). The quantity of outer hair cells (OHCs), IHCs and anti-PSD95-labeled puncta and the density of SGN peripheral fibers (fiber density) near IHCs were measured after respective immunofluorescence-labeling. The ratio of the number of OHCs to IHCs number (OHC/IHC ratio) and the ratio of the number of PSD95 puncta number to IHCs (PSD95/IHC ratio) was used to evaluate the quantity of hair cells and IHC synapses, respectively. After 8 h or 24 h, there was no statistical difference in the OHC/IHC ratio, fiber density and PSD95/IHC ratio among the non-ES, 50 and 100 μA groups (*P* values in Table 1 and Figures 3A–C). After 48 h, PSD95/IHC ratio of 48 h/50 μA group were also comparable to that of non-ES group (*P* = 0.9170, Figures 3C,F,J), but the fiber density was less than that in non-ES group (*P* = 0.0097, Figures 3B,E,G,I,K). Compared to with non-ES explants, cochlear explants electrically stimulated with a 100 μA pulse for 48 h showed significantly decreased fiber density and PSD95/IHC ratio (*P* < 0.0001, Figures 3B,C,M–O). However, after 24 h or 48 h, the OHC/IHC ratio in explants treated with 50 μA or 100 μA ES was still comparable to that in non-ES explants (24 h/50 μA group *P* > 0.9999, 24 h/100 μA group *P* = 0.5872, 48 h/50 μA group *P* = 0.6174, 48 h/100 μA group *P* = 0.3631, respectively when compared with non-ES group, Figure 3A). Additionally, there was no obvious difference between the hair cell morphology of ES explants and non-ES explants (Figures 3D,H,L).

**TABLE 1:** *P* value of OHC/IHC ratio, fiber density and PSD95/IHC ratio of the 50 and 100 μA group compared with Non-ES group.

	**8 h**		**24 h**		**48 h**	
	**50 μA**	**100 μA**	**50 μA**	**100 μA**	**50 μA**	**100 μA**
OHC/IHC	0.8955	0.4851	>0.9999	0.5872	0.6174	0.3631
PSD95/IHC	0.4526	0.7005	0.5011	0.3921	0.9170	<0.0001
Fiber Density	0.9096	0.8528	0.4702	0.4854	0.0097	<0.0001

*OHC/IHC, the ratio of OHC number to IHC number; PSD95/IHC, the ratio of PSD95 puncta number to IHC number.*

### The Quantity of IHC Synapses and SGN Peripheral Fibers Decreased Synchronously Under ES

**FIGURE 4:**
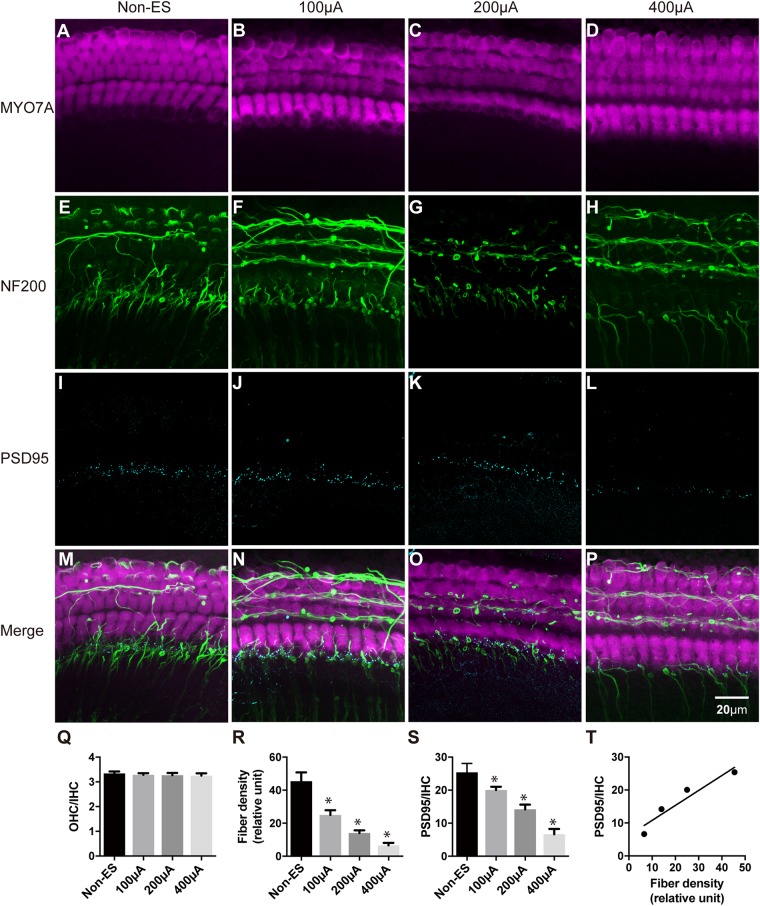
The density of SGN peripheral fibers and the quantity of IHC synapses were synchronously decreased with increasing ES intensity. The quantity and morphology of hair cells (in magenta) were comparable in explants treated with non-ES, 100 μA ES, 200 μA ES or 400 μA ES for 48 h (**A–D**; *P* = 0.6957, *P* = 0.5289, *P* = 0.3364, compared with non-ES in panel **Q**, one-way ANOVA followed by Dunnett’s multiple comparisons test, the following comparisons in this paper used the same method unless otherwise noted, *n* = 5 in each group). The density of SGN peripheral fibers (in green) in explants treated with 100, 200, and 400 μA ES for 48 h was significantly less than that in non-ES explants (**E–H**; ^∗^ in panel **R**, *P* = 0.0001 when compared to non-ES, *n* = 20 in each group). The PSD95/IHC ratio in explants treated with 100, 200, and 400 μA ES for 48 h was also significantly less than that in non-ES explants (**I–L**; ^∗^ in panel **S**, *P* = 0.0001 when compared to non-ES, *n* = 20 in each group). **(M–P)** The merged images of the upper three images under the same conditions, respectively. **(T)** There was a significant correlation between the change of SGN peripheral fiber density and the PSD95-punch/IHC ratio with the increase of ES intensity (Pearson test, *r* = 0.9538, *P* = 0.0462). Data represent the mean + SEM.

We further used higher intensities of biphasic charge-balanced pulses to stimulate the cochlear explants for 48 h. Compared to the non-ES group with PSD95/IHC ratio counting to 25.38, PSD95/IHC ratios of 100, 200, and 400 μA groups significantly decreased to 20.06, 14.21, and 6.64, respectively (Figure 4S). Additionally, the fiber densities of 100, 200, and 400 μA groups also significantly decreased to 4.17, 2.34, and 1.10, respectively, compared to 7.58 in the non-ES group (Figure 4R). The density of SGN peripheral fibers and the quantity of IHC synapses were synchronously decreased with increasing ES intensity (Figures 4E–P). However, there was still no significant difference in the morphology of hair cells and the OHC/IHC ratios among these groups (Figures 4A–D,Q). There was a significant correlation between fiber density and PSD95/IHC ratio (Pearson test, *r* = 0.954, *P* = 0.046, Figure 4T). These results demonstrated that ES synchronously decreased the quantity of IHC synapses and SGN peripheral fibers in a current intensity-dependent manner, but did not change the morphology or quantity of hair cells. Thus, we only used fiber density to evaluate the change of cochlear structure in the following experiments.

### Inhibition of Calcium Influx Attenuated the ES-Induced Loss of SGN Peripheral Fibers and IHC Synapses

**FIGURE 5:**
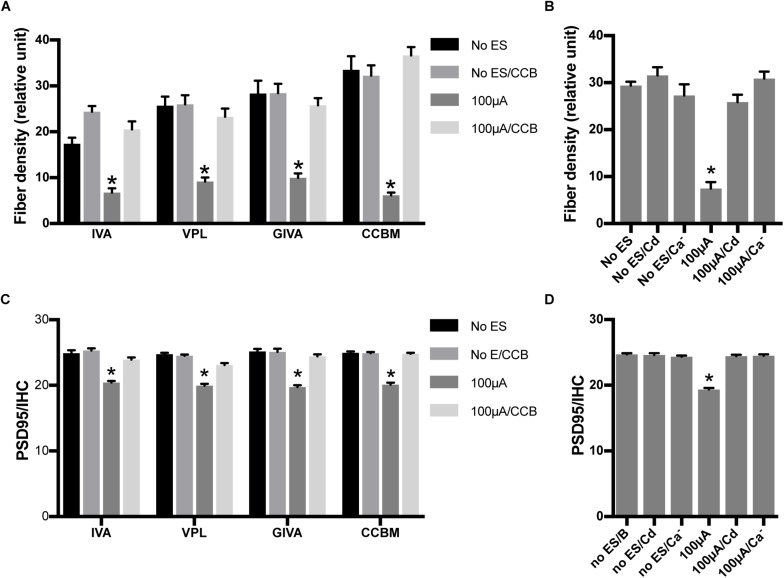
Inhibiting calcium influx attenuated the loss of SGN peripheral fibers and IHC synapses induced by ES. **(A)** PSD95/IHC ratio was significantly decreased in explants treated with 100 μA/48 h ES compared with non-ES explants, while explants simultaneously treated with 100 μA/48 h ES and various types of voltage-dependent calcium channel blockers (CCB), including 10 μM VPL, 1 μM GVIA, 1 μM IVA and their mixture (CCBM), were not significantly different from the non-ES group *n* = 7–12 in each group. **(B)** When ES-treated explants were also treated with 10 μM Cd or maintained in calcium-free medium (Ca^–^), the PSD95/IHC ratio was also comparable to that of non-ES explants *n* = 9 in each group. **(C)** The density of SGN peripheral fibers was comparable in ES-treated explants also treated with VPL, GVIA, IVA or CCB and in non-ES explants, *n* = 12 in each group. **(D)** The density of SGN peripheral fibers was also comparable in ES-treated cochlear explants also treated with Cd or maintained in calcium-free medium and in non-ES explants, *n* = 12 in each group. **^∗^***p* < 0.001 compared with any other group in the same experiment, one-way ANOVA followed by Dunnett’s multiple comparisons. Data represent the mean + SEM.

To investigate the role of calcium influx through VDCCs in the ES-induced degeneration of SGN peripheral fibers and IHC synapses, we inhibited calcium influx in 48 h/100 μA cochlear explants by bath application of various VDCC blockers, i.e., 10 μM L-type Ca^2+^ channel blocker VPL, 1 μM N-type Ca^2+^ channel blocker GVIA, 1 μM P/Q-type Ca^2+^ channel blocker IVA and their mixture (CCBM). The fiber density and PSD/IHC ratio of 48 h/100 μA group was significantly lower than those of the non-ES group as described above (*P* < 0.0001). However, fiber density and PSD/IHC ratio of the groups treated with any VDCC blocker were comparable to those of the non-ES group (*P* in Table 2 and Figures 5A,C). We also inhibited calcium influx in 48 h/100 μA cochlear explants by maintaining them in Ca^2+^-free medium or in medium with 10 μM Cd, a non-selective calcium channel blocker. As a result, the fiber density and PSD/IHC ratio were also comparable to those of the non-ES group (*P* in Table 3 and Figures 5B,D). These results suggested that calcium influx through VDCCs is vital to the ES-induced degeneration of SGN peripheral fibers and IHC synapses.

**TABLE 2:** *P* value of fiber density and PSD95/IHC ratio of the Non-ES/CCB and 100 μA/CCB group compared with Non-ES group.

	**PSD95/IHC**	**Fiber Density**
Non-ES/Ca^–^	0.6568	0.8194
Non-ES/Cd	0.9983	0.8073
100 μA/Ca^–^	0.9455	0.9527
100 μA/Cd	0.8361	0.4058

*VPL, verapamil; IVA, ω-agatoxin IVA; GVIA, ω-conotoxin GVIA; CCBM, the mixture of VPL; IVA and GVIA, CCB, calcium channel blockers.*

**TABLE 3:** *P* value of fiber density and PSD95/IHC ratio of the Non-ES/Ca^–^, Non-ES/Cd, 100 μA/Ca^–^ and 100 μA/Cd group compared with Non-ES group.

	**PSD95/IHC**		**Fiber Density**	
	**Non-ES**	**100 μA**	**Non-ES**	**100 μA**
VPL	0.9208	0.0002	0.9995	0.7532
IVA	0.7979	0.2014	0.0041	0.3629
GVIA	0.9995	0.5060	> 0.9999	0.8033
CCBM	0.9969	0.9618	0.9768	0.7404

*Non-ES/Ca^–^, cochlear explants without electrical stimulation in calcium-free medium; Non-ES/Cd, cochlear explants without electrical stimulation in medium with cadmium chloride; 100 μA/Ca^–^, cochlear explants with 100 μA electrical stimulation in calcium-free medium; 100 μA/Cd, cochlear explants with 100 μA electrical stimulation in medium with cadmium chloride.*

### ES Increased the Activity of ROS and RNS in Cochlear Explants

**FIGURE 6:**
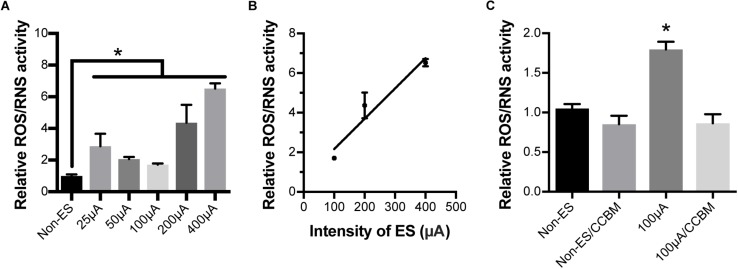
Electrical stimulation increased ROS/RNS activity in cochlear explants but inhibiting calcium influx completely abolished this effect. **(A)** ROS/RNS activity of explants under 25, 50, 100, 200, and 400 μA ES for 48 h was significantly higher than that of non-ES explants. ^∗^*P* < 0.05 when compared with non-ES group, *n* = 3 in each group, measurements were repeated three times. **(B)** ROS/RNS activities increased in an ES intensity-dependent manner. Pearson’s test, *r* = 0.9691, *P* = 0.1587 (two-tailed). **(C)** When 100 μA/48 h ES-treated cochlear explants were also treated with CCB mixture (CCBM), the level of ROS/RNS activity was comparable to that in non-ES explants. ^∗^*p* < 0.001 when compared with any other group, *n* = 3 in each group, measurements were repeated three times. Data represented the mean + SEM.

To investigate whether ES caused oxidative stress in cochlear explants by increasing calcium influx, we measured ROS/RNS activity in explants under various intensities of ES for 48 h. ROS/RNS activity in explants under ES with amplitudes of 25, 50, 100, 200, and 400 μA were increased to 2.9, 2.1, 1.7, 4.4, and 6.5-fold to that of non-ES group, respectively (*P* = 0.0020, 0.0442, 0.1606, <0.0001, and <0.001, respectively when compared with the non-ES group, Figure 6A). In addition, ROS/RNS activity increased in an intensity-dependent manner when cochlear explants had amplitudes greater than 50 μA (Figure 6B). To investigate the role of calcium influx through VDCCs in the change in ROS/RNS activity, we added a mixture of VPL, GVIA and IVA to culture medium of 48 h/100 μA cochlear explants. As a result, ROS/RNS activity decreased to a level comparable to that of the non-ES group (*P* = 0.1072, Figure 6C). These results suggested that ES could increase ROS/RNS activity and cause oxidative stress by increasing calcium influx through VDCCs.

### ES Inhibited GPx Expression in Cochlear Explants

**FIGURE 7:**
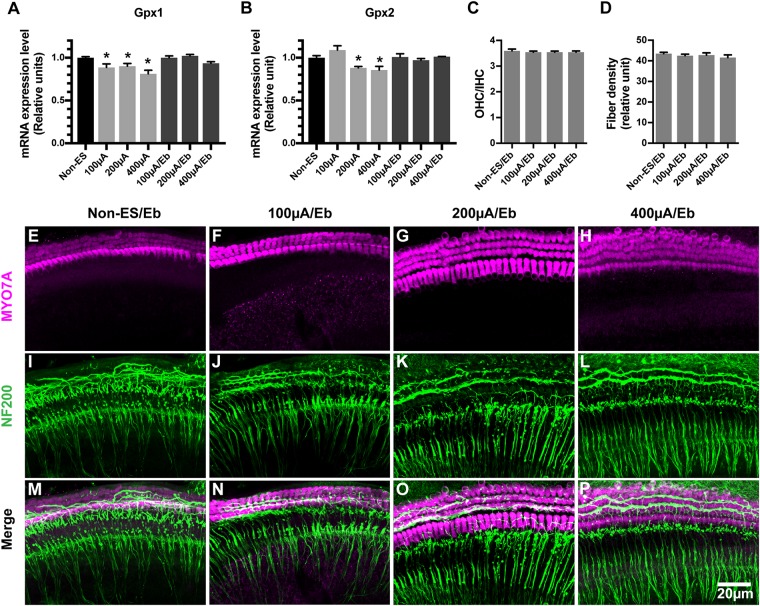
The mRNA expression level of GPx1 and GPx2 in cochlear explants decreased under ES and ebselen abolished this effect and the loss of SGN peripheral fibers. **(A,B)** ES treatment of 200 and 400 μA/48 h both significantly decreased the mRNA expression level of both GPx1 and GPx2 in explants while 100 μA/48 h-ES treatment only decreased the mRNA expression level of GPx1 (^∗^, GPx1: 200 μA, *P* = 0.0231; 400 μA, *P* = 0.0233; GPx2: 200 μA, *P* = 0.0484; 400 μA, *P* = 0.0228, when compared to non-ES group, *n* = 3 in each group, measurements were repeated three times). When the cochlear explants were treated with ES and 40 μM Ebselen at the same time, mRNA expression level of GPx1 and GPx2 was comparable to that in non-ES group (GPx1: 100 μA/Eb, *P* > 0.9999; 200 μA/Eb, *P* = 0.9738; 400 μA/Eb, *P* = 0.4027; GPx2: 100 μA/Eb, *P* > 0.9999; 200 μA/Eb, *P* > 0.9999; 400 μA/Eb, *P* > 0.9999 compared to non-ES group). When the cochlear explants were treated with 40 μM Ebselen, OHC/IHC ratio (**C**, *P* = 0.7997, *P* = 0.7629, *P* = 0.7639, in each group.) and the density of SGN peripheral fibers (**D**, *P* = 0.7860, *P* = 0.9025, *P* = 0.3482, *n* = 20 in each group.) in 100, 200, and 400 μA/48 h-ES groups had no significantly statistical difference from those in the non-ES group. **(E–P)** Representative images showed that there was no significant loss of hair cells (in magenta) and SGN peripheral fibers (in green) in ES-treated explants when they were maintained in medium with 40 μM Ebselen. Data represented the mean + SEM.

We hypothesized that the ES-induced increase in ROS/RNS activity in cochlear explants might be due to the altered expression of oxidative stress-related genes. We evaluated the mRNA expression levels of GPx1 and GPx2 in cochlear explants under various intensities of ES and without ES. Significant decreases in the GPx1 and GPx2 expression levels were both observed in 200 μA/48 h- and 400 μA/48 h-treated explants compared with non-ES explants, respectively (GPx1: 200 μA *P* = 0.0231 and 400 μA *P* = 0.0233, GPx2: 200 μA *P* = 0.0484 and 400 μA *P* = 0.0228, Figures 7A,B). The GPx1 expression level in 100 μA/48 h-treated explants also decreased compared to that in non-ES explants (*P* = 0.0647, Figure 7A). These results demonstrated that ES could result in downregulation of GPx1 and GPx2 mRNA expression levels.

### Ebselen Prevented the Decrease of GPx Expression as Well as the Loss of SGN Peripheral Fibers in Cochlear Explants Exposed to ES

Ebselen is an organoselenium compound that acts as a GPx mimetic and is thereby able to prevent the cellular damage induced by the ROS and RNS generated and accumulated during various cellular processes. To investigate whether the ES-mediated downregulation of GPx and the increase in ROS/RNS activity caused the degeneration of SGN peripheral fibers and IHC synapses, we maintained cochlear explants in medium with 40 μM ebselen for 48 h. As a result, the GPx1 and GPx2 expression levels in 100 μA/48 h-, 200 μA/48 h- and 400 μA/48 h-treated cochlear explants were comparable level to those in non-ES explants (Figures 7A,B). Moreover, the density of SGN peripheral fibers in all ES-treated groups was also comparable to that in non-ES group (Figures 7C–P). These results indicated that ES-induced downregulation of GPx1 and GPx2 expression levels caused the degeneration of SGN peripheral fibers in cochlear explants.

### Increased Oxidative Stress in Cochlear Explants Induced by H_2_O_2_ Treatment Resulted in the Loss of SGN Peripheral Fibers

**FIGURE 8:**
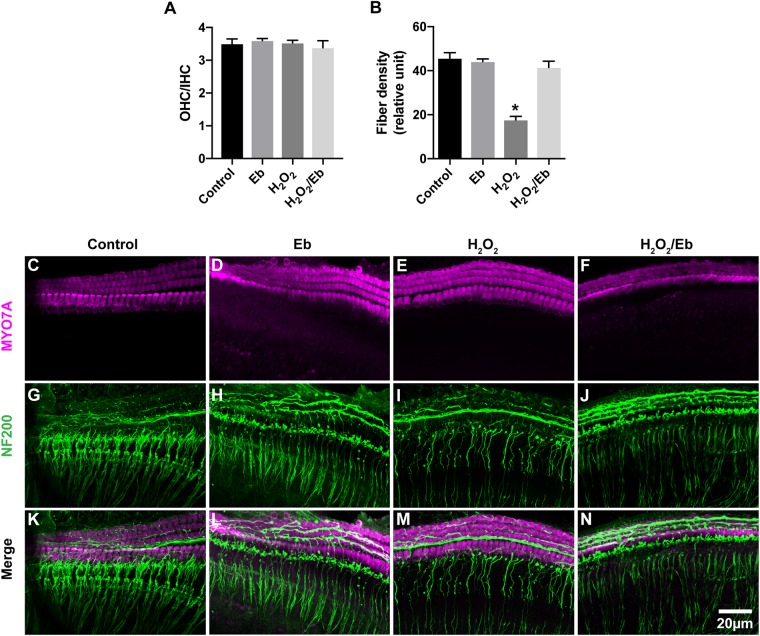
Increased oxidative stress in cochlear explants induced by H_2_O_2_ treatment resulted in the loss of SGN peripheral fibers. **(A)** Treatment of explants with 250 μM H_2_O_2_, 40 μM ebselen or both did not cause any significant difference in the OHC/IHC (in magenta) ratio from that of explants without these treatments. *P* = 0.9990, *P* = 0.9294, *P* = 0.8813, respectively, *n* = 3–5 in each group. **(B)** Treatment of cochlear explants with 250 μM H_2_O_2_ significantly decreased the density of SGN peripheral fibers (in green, ^∗^*P* < 0.0001), while treatment of cochlear explants with both 250 μM H_2_O_2_ and 40 μM ebselen did not decrease the density (*P* = 0.3828, *n* = 8 in each group), compared to the density in explants without H_2_O_2_ or ebselen treatment. **(C–N)** Typical images of cochlear explants treated with 8 h/control **(C,G,K)**, 8 h/Eb **(D,H,L)**, 8 h/H_2_O_2_ **(E,I,M)** and 8 h/H_2_O_2_ Eb **(F,J,N)**. The quantity and morphology of IHCs and OHCs (in magenta, labeled with anti-Myo7A) were comparable in explants treated with control **(C)**, Eb **(D)**, H_2_O_2_ **(E)**, and H_2_O_2_ Eb **(F)**. The density of SGN peripheral fibers (in green, labeled with anti-neurofilament-200, NF200) were similar explants treated with control **(G)**, Eb **(H)**, and H_2_O_2_ Eb **(J)**, while the density was less than that in explants treated with H_2_O_2_ **(I)**. Data represented the mean + SEM.

To further investigate the role of oxidative stress in ES-induced degeneration of SGN peripheral fibers, we added H_2_O_2_ to cochlear explant cultures to induce oxidative stress and evaluated the density of SGN peripheral fibers. Similar to ES, maintaining cochlear explants in medium with 250 μM H_2_O_2_ for 8 h did not change the hair cells morphology and OHC/IHC ratio (*P* = 0.9990, Figures 8A,E) but did significantly decrease the density of SGN peripheral fibers (*P* < 0.0001, Figures 8B,I,M), compared to maintaining cochlear explants without H_2_O_2_ treatment (Figures 8C,G,K). However, the quantity and morphology of HCs and the fiber density of explants simultaneously treated with 250 μM H_2_O_2_ and 40 μM ebselen for 8 h was not different (*P* = 0.3828, Figures 8B,D,F,H,J,L,N), from that of explants without treatment. These results further indicated that oxidative stress could induce the degeneration of SGN peripheral fibers.

## Discussion

Electrical stimulation is used by CI and other neurostimulation devices to activate targeting neurons. The impact of ES on targeted and related neural structures when neurostimulation devices are used as modulators of existing neural function instead of as substitutes of non-functioning neural tissues, warrants additional attention. As shown in cochlea implant recipients using EAS technology, there was a delayed loss of residual low-frequency hearing function (Von Ilberg et al., 1999; Gantz and Turner, 2003; Kiefer et al., 2005). Here we show, that ES could degenerated the connection between the targeted neuron and modulated neural structures *in vitro*. In addition, calcium influx through VDCCs and resulting oxidative stress played important roles in this effect.

Our study suggested that continuous charge-balanced biphasic ES with an intensity up to 48 h/400 μA did not change the numbers of hair cells in cochlear explants. In accordance with our study, a recent *in vitro* study also reported that ES could induce synaptic change in cochlear tissues (Peter et al., 2019). In addition, several previous animal studies also found no morphological changes in hair cells and SGNs associated with ES (Ni et al., 1992; Shepherd et al., 1994; Coco et al., 2007; Irving et al., 2013; O’Leary et al., 2013), even though low-frequency hearing deteriorate after ES (O’Leary et al., 2013; Tanaka et al., 2014). A postmortem histopathological study also suggested that there was no significant loss of SGNs and hair cells in EAS recipients with delayed hearing loss (Quesnel et al., 2015). Our study demonstrated that SGN peripheral fibers and IHC synapses in cochlear explants decreased under the ES with charge-balanced biphasic pulses used by CIs. The charge intensities used in this study were far less than the maximum charge intensities allowed in commercial CIs. However, animal studies are warranted to further investigate whether a similar change is the cause of residual low-frequency hearing loss in EAS recipients.

Electrical stimulation can induce the activation of VDCCs and result in Ca^2+^ influx. Calcium influx through VDCCs was involved in the inhibition of SGN neurite extension induced by continuous ES or membrane depolarization accomplished by raising extracellular K^+^ (Roehm et al., 2008; Shen et al., 2016). Calcium overload has been shown to cause damage to SGNs (Hegarty et al., 1997; Roehm et al., 2008). Our study suggested that blocking various types of VDCCs by bath application of VDCC blockers, by the non-selective VDCC blocker cadmium or by the removal of extracellular Ca^2+^ attenuated the ES-induced loss of SGN peripheral fibers and IHC synapses. The mixture of VPL, GVIA, and IVA also abolished the ES-induced increase in ROS/RNS activity in cochlear explants. These results suggest that calcium influx through VDCCs plays a key role in ES-induced cochlear synaptic degeneration.

The ES-induced loss of SGN peripheral terminals and IHC synapses with the preservation of hair cells and SGNs is similar to the changes that appeared in the early stage of noise-induced hearing loss (Kujawa and Liberman, 2009; Lin et al., 2011). Previous studies have suggested that the excitotoxicity and calcium overload play critical roles in noise-induced hearing loss (Le Prell et al., 2007; Kujawa and Liberman, 2009). Mimicking excitotoxicity in cochlear explant culture by brief treatment with NMDA and kainite also resulted in the loss of IHC synapses and SGN peripheral axons with the organ of Corti and SGNs intact (Wang and Green, 2011). Taken together, these findings suggest that the manifestations of cochlear explants under ES were similar to the findings in animal studies of CI chronic ES, and noise-induced hearing loss and in the *in vitro* study of excitotoxicity in cochlear explants. This suggested that excitotoxicity and calcium overload might play important roles in delayed EAS hearing loss. This theory was supported by our results that the inhibition of calcium influx prevented the loss of IHC synapses and SGN peripheral terminals. Interestingly, a close correlation between EAS hearing loss and a history of noise-induced hearing loss shown in a recent clinical study provides further support for this postulation (Kopelovich et al., 2014).

Our study showed that ES induced an increase in ROS/RNS activity in cochlear explants. The increase in ROS/RNS activity was closely correlated with the intensity of ES. After the increase in ROS/RNS activity was prevented by ebselen, the loss of SGN peripheral fibers in ES-treated cochlear explants was significantly attenuated to a level comparable to that of non-ES cochlear explants. These results suggested that oxidative stress played an important role in the ES-induced loss of SGN-IHC connections. Oxidative stress has also been reported to play important roles in hearing loss induced by noise, aminoglycoside antibiotics, cisplatin and aging (Choi and Choi, 2015; Sheth et al., 2017; Tavanai and Mohammadkhani, 2017). Excessively high ROS and RNS activity can cause damage to DNA, lipids and proteins, trigger hair cell death and result in hearing loss (Fetoni et al., 2015). We added H_2_O_2_ to the culture medium to induce oxidative stress and consequently caused a change similar to the ES-induced loss of IHCs-SGNs connection. These results further supported our hypothesis that ES induces cochlear synaptic degeneration through calcium influx-induced oxidative stress.

This study demonstrated that GPx1 and GPx2 expression levels significantly decreased after 200 μA/48 h and 400 μA/48 h ES. Interestingly, GPx1 expression level significantly decreased even after a relatively weak ES, i.e., 100 μA/48 h of ES, while GPx2 expression level insignificantly decreased. In accordance with our study, a decrease in GPx1 activity was shown to play an important role in noise-induced hearing loss (Kil et al., 2007). The targeted mutation of the GPx1 gene in mice also increased their vulnerability to noise-induced hearing loss (Ohlemiller et al., 2000). Ebselen could inhibit iNOS (Zembowicz et al., 1993) and mimic the anti-oxidative enzyme GPx (Ohlemiller et al., 2000). Ebselen treatment reducse the severity and duration of noise-induced hearing loss of in animals as well as human beings (Pourbakht and Yamasoba, 2003; Kil et al., 2017). In our study, ebselen treatment significantly increased GPx1 and GPx2 expression levels which were decreased by ES. Additionally, the ES-induced loss of SGN peripheral fibers was completely abolished. These results strongly supported that the decrease in GPx1 and GPx2 expression levels played a vital role in ES-induced loss of IHC-SGN connections. Our study also indicated that ebselen might be a promising agent to protect the residual hearing of EAS recipients although further *in vivo* studies are needed.

In conclusion, our study demonstrated that ES with charge-balanced biphasic pulses could result in the synchronous degeneration of SGN peripheral fibers and IHC synapses in a current intensity- and duration-dependent manner *in vitro*. Calcium influx through VDCC and resulting oxidative stress played key roles in this effect. Ebselen was shown to be a potential protector of ES-induced cochlear synaptic degeneration. Our study provides novel insights into delayed hearing loss in EAS recipients as well as the impact of other neurostimulation devices on targeting neural structures. However, only middle turn of immature cochlea was used in our study. Whether there is different impact of electrical stimulation on different part of cochlea or mature cochlear tissues should be investigated further. Notably, animal studies are also necessary to investigate the status of IHC synapses and SGN peripheral fibers under chronic ES.

## Data Availability Statement

All datasets generated for this study are included in the manuscript/supplementary files.

## Ethics Statement

This study was carried out in accordance with the recommendations of the Ethics Review Board of Eye and ENT Hospital of Fudan University. The protocol was approved by the Ethics Review Board of Eye and ENT Hospital of Fudan University.

## Author Contributions

NS contributed the experiments of duration and intensity effect, calcium influx manipulation as well as related statistical analysis. QL performed all other experiments and related analysis. BL contributed a part of imaging work under confocal microscope. ZW contributed the design of experiments. SL contributed the design of all experiments, the writing of manuscript and preparation of figures. CX and ZS contributed the adjust of multichannel charge-balanced biphasic pulse generator.

## Conflict of Interest

The authors declare that the research was conducted in the absence of any commercial or financial relationships that could be construed as a potential conflict of interest.
